# Expression and Clinical Role of Protein of Regenerating Liver (PRL) Phosphatases in Ovarian Carcinoma

**DOI:** 10.3390/ijms12021133

**Published:** 2011-02-11

**Authors:** Reuven Reich, Shany Hadar, Ben Davidson

**Affiliations:** 1 Institute of Drug Research, School of Pharmacy, Faculty of Medicine, The Hebrew University of Jerusalem, Jerusalem 91120, Israel; E-Mail: shanzelize81@gmail.com; 2 Division of Pathology, Norwegian Radium Hospital, Oslo University Hospital, N-0310 Oslo, Norway; 3 The Medical Faculty, University of Oslo, N-0316 Oslo, Norway

**Keywords:** ovarian carcinoma, effusion, metastasis, PRL phosphatases, survival

## Abstract

The present study analyzed the expression and clinical role of the protein of regenerating liver (PRL) phosphatase family in ovarian carcinoma. *PRL1-3* mRNA expression was studied in 184 tumors (100 effusions, 57 primary carcinomas, 27 solid metastases) using RT-PCR. PRL-3 protein expression was analyzed in 157 tumors by Western blotting. *PRL-1* mRNA levels were significantly higher in effusions compared to solid tumors (*p* < 0.001), and both *PRL-1* and *PRL-2* were overexpressed in pleural compared to peritoneal effusions (*p* = 0.001). PRL-3 protein expression was significantly higher in primary diagnosis pre-chemotherapy compared to post-chemotherapy disease recurrence effusions (*p* = 0.003). *PRL-1* mRNA expression in effusions correlated with longer overall survival (*p* = 0.032), and higher levels of both *PRL-1* and *PRL-2* mRNA correlated with longer overall survival for patients with pre-chemotherapy effusions (*p* = 0.022 and *p* = 0.02, respectively). Analysis of the effect of laminin on PRL-3 expression in ovarian carcinoma cells *in vitro* showed dose-dependent PRL-3 expression in response to exogenous laminin, mediated by Phospholipase D. In contrast to previous studies associating PRL-3 with poor outcome, our data show that PRL-3 expression has no clinical role in ovarian carcinoma, whereas *PRL-1* and *PRL-2* expression is associated with longer survival, suggesting that PRL phosphatases may be markers of improved outcome in this cancer.

## 1. Introduction

Events in cancer progression and metastasis remain less well-defined than those involved in oncogenesis, despite the fact that tumor metastasis is the major factor causing cancer mortality. The indication that the altered regulation of specific protein tyrosine phosphorylation events and signaling pathways may characterize metastatic cells is exciting and may be the basis for new anticancer strategies that would inhibit tumor growth and metastasis.

Protein tyrosine phosphatases (PTP) and tyrosine kinases regulate vital intracellular signaling pathways, controlling most cellular activities [1,2]. Aberrant expression and activation of these enzymes are often causative factors in cancer [3,4].

Protein of regenerating liver-3 (PRL-3) and two closely-related PTPs, PRL-1 and PRL-2, comprise a novel sub-class (the PRL-PTPs) within the large PTP super-family. PRL-1 was identified as an immediate-early gene expressed in mitogen-stimulated cells and regenerating liver [5] and later shown to be a PTP [6]. Subsequently, PRL-2 and PRL-3 were discovered and were shown to share high homology in their amino acid sequence with PRL-1 and to have PTP activity [7–9].

Several recent observations showed that PRL phosphatases, especially PRL-3, may play causal roles in growth regulation, proliferation, cell motility, and invasion [10–12]. Saha *et al.* first linked PRL-3 with the metastasis of human cancer, reporting that elevated PRL-3 expression was detected in all liver metastases from colorectal cancer [13]. Accumulated evidence further demonstrated that PRL-3 is associated with metastasis of multiple tumor types [12,14–19], and has been validated as a potential therapeutic target for metastasis.

Ovarian carcinoma (OC), the most lethal gynecological cancer, ranks fifth in cancer related-deaths among women and is frequently referred to as the “silent killer”, with the majority of patients diagnosed with advanced-stage (FIGO stages III–IV) disease [20]. We previously reported that expression of the phosphatase PAC-1 in OC effusions is associated with poor outcome [21].

In the present study, we examined the expression of PRLs in primary OC, solid metastases and pleural and peritoneal effusions. In view of our previous observation that dual-specificity phosphatases (DUSP), negative regulators of the mitogen-activated protein kinase (MAPK) family, are affected by laminin signaling [22], we additionally investigated the relationship between PRL-3 and exposure of OC cells to laminin *in vitro*. Our results show that PRL-1 mRNA levels are higher in effusions compared to solid tumors and that both PRL-1 and PRL-2 are more highly expressed in pleural compared to peritoneal effusions. Nevertheless, higher PRL-1 and PRL-2 expression in effusions is associated with longer overall survival. In contrast to previous studies in which PRL-3 has been shown to be a marker of poor prognosis in other malignancies, our data suggest that its expression in OC has no clinical role.

## 2. Materials and Methods

### 2.1. Tumors and Patients

The material analyzed in the present study consisted of 100 effusions (72 peritoneal, 28 pleural), 57 primary carcinomas, and 27 solid metastases, submitted for routine diagnostic purposes to the Division of Pathology, Norwegian Radium Hospital, in the period 1996–2005. Effusions were tapped from 90 patients, of whom 81 had one effusion, eight had two effusions, and one had three effusions. Effusions were obtained from patients diagnosed with OC (*n* = 74), predominantly of the serous type, primary peritoneal serous carcinoma (*n* = 11) or tubal serous carcinoma (*n* = 5). Due to their closely-linked histogenesis and phenotype, all these tumors are referred to as OC in the following sections. Primary carcinomas and solid metastases were each a single lesion from each patient. The latter were predominantly omental lesions. The majority of tumors from different sites were not patient-matched. Clinicopathologic data are summarized in Table 1.

Effusions were all received in the fresh non-fixed state immediately after tapping. Specimens were centrifuged and pellets were fresh-frozen at −70 °C in RPMI 1640 medium supplemented with 50% fetal calf serum and 20% dimethyl sulfoxide at a ratio of 1:1, immediately after tapping. Smears and H&E-stained cell block sections were reviewed by a surgical pathologist experienced in cytopathology (BD). Diagnoses were established using morphology and immunohistochemistry.

OC biopsies were similarly snap-frozen. Frozen sections from all tumors were evaluated for the presence of a >50% tumor component and absence of necrosis. H&E-stained sections from these tumors were reviewed to establish tumor type and histological grade.

### 2.2. RT-PCR Analysis

Total RNA was extracted using a commercial kit (Tri Reagent; Sigma-Aldrich, St. Louis, MO, U.S.), and 0.5 μg of total RNA were reverse-transcribed using M-MLV Reverse Transcriptase (Promega, Madison, WI, U.S.) with incubation for 2 h at 37 °C, followed by 5 min at 95 °C, and diluted 1:5 with RNase-free water. Reverse-transcriptase polymerase chain reaction (RT-PCR) analysis was performed on cDNA samples with a DNA thermal cycler (Eppendorf Mastercycler gradient; Eppendorf, Hamburg, Germany) using primer sets detecting PRL and 28S ribosomal RNA. Primer sequences and cycle parameters are detailed in Tables 2 and 3, respectively. RNA from HT-1080 cells served as internal controls.

Products were separated on 1.5% agarose gels, isolated using the Invisorb^®^ Spin DNA extraction kit (Invitek GmbH, Berlin, Germany) and sequenced. Gels were photographed by the KODAK EDAS 290 system. Densitometer analysis of films was performed using a computerized image analysis (NIH IMAGE 1.63) program. *PRL-1*, *PRL-2* and *PRL-3* mRNA levels were established by calculating the target molecule/28S ratio (all cases scored for band intensity compared to control) [24]. Expression intensity of ≤5% of control levels was interpreted as negative. Measurements were made in the linear phase of the reaction.

### 2.3. Western Blotting

Cells and tissues were lysed in 1% NP-40, 20 mM Tris HCl (pH 7.5), 137 mM NaCl, 0.5 mM EDTA, 10% glycerol, 1% protease inhibitor cocktail (Sigma-Aldrich) and 0.1% SDS. Following centrifugation, the supernatant was collected and protein content was evaluated by the Bradford assay. 30 μg from each sample under reducing conditions were loaded into each lane and separated by electrophoresis on a 12% SDS polyacrylamide gel. Following electrophoresis, proteins were transferred to Immobilon transfer membranes (Millipore, Billerica, MA, U.S.). Nonspecific binding was blocked by incubation with TBST (10 mM Tris-HCl (pH 8.0), 150 mM NaCl, 0.1% Tween 20) containing 5% skim milk for 1 h at room temperature. Membranes were subsequently incubated with antibodies against α-tubulin (Sigma-Aldrich) and PRL-3 (Genesis Biotech Inc., Taiwan). Antibody was detected using peroxidase-conjugated AffiniPure goat anti-rabbit IgG or goat anti-mouse IgG (Jackson ImmunoResearch, West Grove, PA, U.S.) and enhanced chemiluminescence Western blotting detection reagents (Thermo Fisher Scientific, Waltham, MA, U.S.). HT-1080 cells served as controls.

### 2.4. Cell Lines and Culture Conditions

The ES-2, OVCAR-8, A2780 and OVCAR-3 OC cell lines were used in this study. ES-2 and OVCAR-3 cells were cultured in DMEM 4.5 GR l^−1^ D-Glucose, while OVCAR-8 and A2780 cells were cultured in RPMI, all supplemented with 10% fetal calf serum, penicillin, essential amino acids and streptomycin (Biological Industries, Beit-Haemek, Israel).

### 2.5. Regulation of PRL-3 Expression

The ES-2, OVCAR-8, A2780 and OVCAR-3 OC cell lines were exposed to various concentrations of laminin (1–20 μg/mL) in the presence of 1-propanol or 2-propanol (0.5%) for 6 h and the protein level of PRL-3 was evaluated by Western blot analysis.

### 2.6. Statistical Analysis

Data were analyzed using the SPSS-PC package, version 16.0 (Chicago, IL, U.S.) comparative analyses of results in effusions, primary and solid metastases using PCR and Western Blot were performed using the Kruskal-Wallis *H* test (analysis of all three sites) or Mann-Whitney test (for analysis of two sites).

PRL expression levels in effusions were analyzed for association with clinicopathologic parameters and survival. Clinicopathologic parameters were grouped as follows: Age: ≤60 *vs.* >60 years; grade: 1–2 *vs.* 3; effusion site: peritoneal *vs.* pleural; FIGO stage: IIIc *vs.* IV; chemotherapy status: pre- *vs.* post-chemotherapy specimens; residual disease volume (≤1 cm *vs.* >1 cm); response to chemotherapy for primary disease and for disease recurrence: complete *vs.* partial response/stable disease/progression. Progression-free and overall survival (PFS; OS) were calculated from the date of the last chemotherapy treatment/diagnosis to the date of recurrence/death or last follow-up, respectively. Univariate survival analyses of PFS and OS were executed using the Kaplan-Meier method and log-rank test. Expression categories in survival analyses were clustered as higher *vs.* lower than median expression. Multivariate analyses of OS were performed using the Cox proportional hazard model.

## 3. Results

### 3.1. PRL Enzyme Expression at Various Anatomic Sites

Previous studies have shown involvement of PRL enzymes in tumor progression of different malignancies. Our objective was to investigate whether PRLs are expressed in OC and to analyze potential anatomic site-related differences in their expression level.

**Figure 1.**
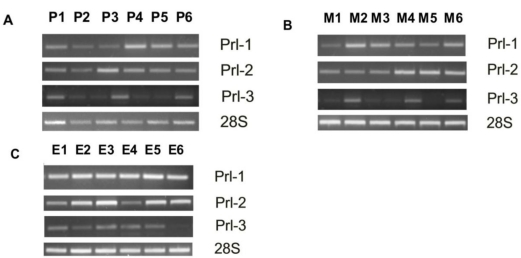

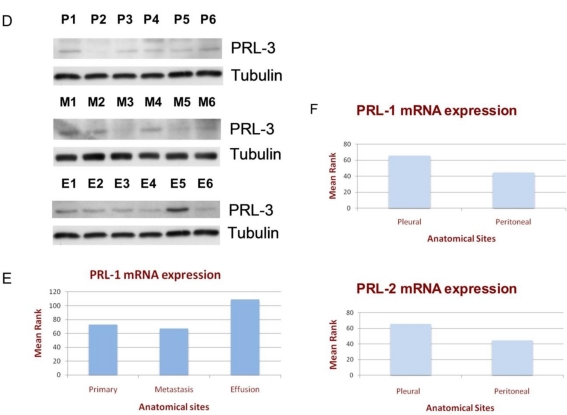
PRL expression at different anatomic sites in ovarian carcinoma (OC). (**A**–**C**) Representative gels showing *PRL-1*, *PRL-2* and *PRL-3* mRNA expression by RT-PCR; (**D**) PRL-3 protein expression by Western blotting; P = primary carcinoma, M = solid metastasis, E = effusion; (**E**) Differential expression of *PRL-1* mRNA in OC effusions compared to primary carcinomas and solid metastases (Kruskal-Wallis H Test, *p* < 0.001); (**F**) Differential expression of *PRL-1* and *PRL-2* mRNA in pleural compared to peritoneal effusions (Mann-Whitney U Test, *p* = 0.001).

*PRL-1* and *PRL-2* mRNA was detected in all 100 effusions and 27 solid metastases and in 56/57 primary carcinomas (Figures 1A, B). *PRL-3* mRNA was detected in all effusions and solid metastases and in 53/57 primary carcinomas (Figure 1C). PRL-3 protein was found in 86/97 effusions, 33/41 primary carcinomas and 16/19 solid metastases (Figure 1D). Comparative analysis of expression levels obtained in quantitative analysis of band size and intensity showed significantly higher *PRL-1* mRNA expression in effusions compared to solid tumors (*p* < 0.001; Figure 1E). *PRL-2* and *PRL-3* mRNA and PRL-3 protein expression was comparable at all anatomic sites (*p* > 0.05; data not shown). Additionally, *PRL-1* and *PRL-2* mRNA expression was significantly higher in pleural compared to peritoneal effusions (*p* = 0.001 for both; Figure 1F), with a similar trend for *PRL-3* mRNA (*p* = 0.085) and PRL-3 protein (*p* = 0.054).

### 3.2. The Clinical Significance of PRL Enzyme Expression

*PRL* mRNA and PRL-3 protein expression levels were analyzed for association with clinicopathologic parameters. No significant associations were observed between PRL mRNA expression and chemotherapy status, patient age, histological grade, FIGO stage, residual disease volume or response to chemotherapy at diagnosis or first disease recurrence. However, PRL-3 protein expression was higher in pre-chemotherapy compared to post-chemotherapy effusions (*p* = 0.003), a finding that was significantly related to previous treatment with platinum agents (*p* = 0.024), but was only as a trend with respect to paclitaxel treatment (*p* = 0.056). PRL-3 protein expression was unrelated to the other clinicopathologic parameters (*p* > 0.05).

Survival data were available for 49 of the 57 patients with primary OC. The follow-up period for these patients ranged from 3–91 months (mean = 32 months, median = 31 months). At last follow-up, 20 patients were alive without disease, eight were alive with disease and 20 were dead of disease. One patient was lost to follow-up. PFS ranged from 0-65 months (mean = 16 months, median = 10 months). *PRL1-3* mRNA and PRL-3 protein expression in primary OC were unrelated to OS or PFS in this cohort (*p* > 0.05; data not shown).

**Figure 2.**
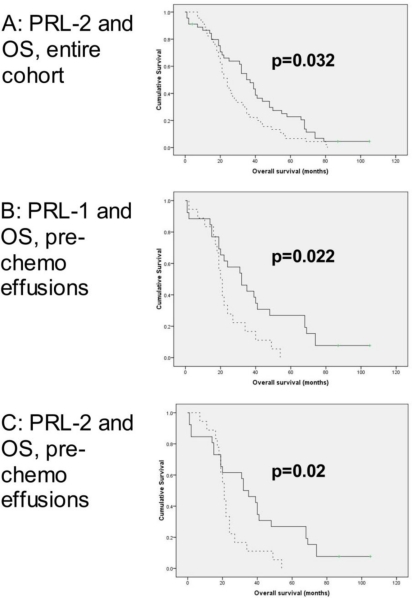
*PRL-1* and *PRL-2* mRNA expression in OC effusions is associated with longer overall survival (OS). (**A**) Kaplan-Meier survival curve showing the association between *PRL-2* mRNA expression and OS in the entire effusion cohort (*n* = 90). Patients with effusions having higher than median *PRL-2* mRNA levels (*n* = 45; solid line) had mean OS of 39 months compared to 29 months for patients with lower than median expression levels (*n* = 45, dashed line; *p* = 0.032); (**B**) Kaplan-Meier survival curve showing the association between *PRL-1* mRNA expression and OS for women with pre-chemotherapy effusions (*n* = 44). Patients with effusions having higher than median *PRL-1* mRNA levels (*n* = 26; solid line) had mean OS of 39 months compared to 23 months for patients with lower than median expression levels (*n* = 18, dashed line; *p* = 0.022); (**C**) Kaplan-Meier survival curve showing the association between *PRL-2* mRNA expression and OS for women with pre-chemotherapy effusions (*n* = 44). Patients with effusions having higher than median *PRL-2* mRNA levels (*n* = 26; solid line) had mean OS of 39 months compared to 24 months for patients with lower than median expression levels (*n* = 18, dashed line; *p* = 0.02).

Survival data were available for all 90 patients with OC effusions. The follow-up period for these patients ranged from 1–105 months (mean = 34 months, median = 27 months). At last follow-up, one patient was alive without disease, one was alive with disease and 87 were dead of disease. One patient died of unrelated causes. PFS ranged from 0–82 months (mean = 8 months, median = 5 months). In univariate survival analysis of the entire cohort, higher *PRL-2* mRNA expression was associated with longer OS (*p* = 0.032; Figure 2A), with a similar trend for *PRL-1* (*p* = 0.107) and PRL3 protein (*p* = 0.105). *PRL-3* mRNA expression was unrelated to survival.

In univariate survival analysis of patients with primary diagnosis pre-chemotherapy effusions, higher levels of both *PRL-*1 and *PRL-2* mRNA were significantly associated with longer OS (*p* = 0.022 and *p* = 0.02, respectively; Figures 2B,C), with a similar trend for *PRL-3* (*p* = 0.093). PRL3 protein expression was unrelated to survival (*p* > 0.05).

In univariate survival analysis of patients with disease recurrence post-chemotherapy effusions, none of the studied molecules was significantly associated with OS.

PRL expression in effusions was unrelated to PFS in any of these patient groups.

Multivariate Cox analysis of OS was performed for the entire effusion cohort, as well as for patients with primary diagnosis effusions. The parameters entered into the former analysis consisted of *PRL-1* and *PRL-2* mRNA expression, PRL-3 protein expression and the two clinical parameters that were significantly associated with OS in univariate analysis, response to chemotherapy at diagnosis (*p* < 0.001) and at first disease recurrence (*p* = 0.001). Only chemoresponse at diagnosis (*p* = 0.009) and at first disease recurrence (*p* = 0.001) was an independent prognostic marker.

The parameters entered in the multivariate Cox analysis of OS for patients with primary diagnosis effusions consisted of *PRL-1*, *PRL-2* and *PRL-3* mRNA expression, as well as residual disease volume (*p* = 0.163) and response to chemotherapy at diagnosis (*p* = 0.004) and at first disease recurrence (*p* = 0.021). Similarly to the entire cohort, only chemoresponse at diagnosis (*p* = 0.016) and at first disease recurrence (*p* = 0.01) was an independent prognostic marker.

### 3.3. PRL-3 Regulation

**Figure 3.**
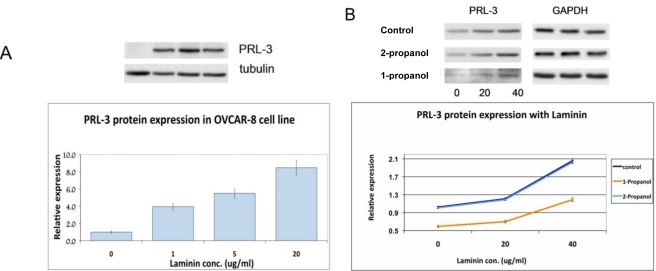
PRL-3 expression in response to laminin stimulation. (**A**) OVCAR-8 OC cells were exposed to different concentrations of exogenous laminin for 6 h and protein expression evaluated; (**B**) PRL-3 expression in presence of primary and secondary alcohols. OVCAR-8 OC cells were exposed to various laminin concentrations in the presence of 1-propanol and 2-propanol and protein expression evaluated.

The molecular mechanism and regulation of PRL-3 is not clear yet. It has been suggested to be involved in several intracellular pathways regulating cellular proliferation and tumor dissemination. Our previous studies have shown a direct correlation between cellular phosphatases and laminin signaling in malignant melanoma [22]. Therefore, we examined the effect of exogenous laminin on the expression of PRL-3 protein in OC cells *in vitro*. Our results show a significant dose-dependent expression of PRL-3 in response to exogenous laminin (Figure 3A). In previous studies, we have shown that laminin signaling involves activation of phospholipase D (PLD) enzymes [25,26]. We repeated the experiments in the presence of primary and secondary alcohol, known modulators of PLD signal transduction. Indeed, 1-propanol inhibited the laminin-induced PRL-3 protein expression while 2-propanol did not show any effect on the expression of the protein, indicating the involvement of PLD in this process (Figure 3B).

## 4. Discussion

The value of PRL-3 as a potential therapeutic target in cancer has been evaluated in different malignancies in recent years, with the majority of studies focusing on metastatic disease [12,14,18,27–29]. The PRL PTPs exhibit different tissue expression levels and patterns. In normal human adult tissues, PRL-3 is expressed predominantly in the heart and striated and smooth muscle cells, with lower expression in the pancreas, and this expression pattern is distinct from the wider expression of PRL-1 and PRL-2 in brain, liver, and kidney [10].

Analysis of genes expressed in normal colorectal epithelia, primary colorectal carcinomas, and liver metastases revealed that PRL-3 expression was lower in primary tumors compared to metastases and essentially undetectable in normal colorectal epithelia, suggesting that elevated levels of PRL-3 contribute to or are a consequence of tumor progression [13].

In contrast to the above-mentioned studies of various solid tumors, in which PRL-3 has been shown to be associated with poor prognosis, our results show that *PRL-3* mRNA and PRL-3 protein expression have no clinical role in OC. Furthermore, PRL-3 protein expression is downregulated along tumor progression, as evidenced by the lower expression of this protein in disease recurrence compared to primary diagnosis effusions. A possible explanation for this phenomenon could be the unique form of OC tumor progression. We recently showed that OC effusions deviate from the epithelial-to-mesenchymal (EMT)-like processes present in most solid tumors by presenting partial mesenchymal-to-epithelial (MET)-like characteristics [30].

Of special interest is the significantly higher expression of *PRL-1* mRNA in OC effusions compared to both primary carcinomas and solid metastases, suggesting that this phosphatase has an important regulatory role at this anatomic site. Further, we found a correlation between both *PRL-*1 and *PRL-2* mRNA levels in OC effusions and longer OS, supporting the relevance of this family for OC effusion biology. To our best knowledge, this is the first description of such a correlation. The fact that this finding did not reach significance at multivariate analysis owes at least to some extent to the strong clinical role of chemoresponse in our cohort and in OC in general.

As shown by previous studies, PRL-3 is a multitasking phosphatase involved in modulating cancer metastasis. However, the mechanisms of PRL-3 action and regulation are poorly understood since neither the substrates nor the signaling pathways have been characterized. It was shown *in vitro* that PRL-3 might act as an upstream regulator of the phosphatase and tensin homologue deleted on chromosome 10 (PTEN)-phosphoinositide 3-kinase (PI3K) signaling network [31]. Others have shown PRL-3 association with the integrin β1-ERK1/2 phosphorylation pathway [32]. Recently, it has been suggested that PRL-1 and PRL-3 may downregulate p53 via the activation of PIRH2 and MDM2 [33].

Our group has not previously studied the molecular interactions of PRL in cancer. However, we previously reported on the downregulation of the DUSP family member MKP-1, with concomitant upregulation of PAC-1, another DUSP member, in A375SM melanoma cells following exposure to the extracellular protein laminin [22], leading us to hypothesize that laminin may regulate other phosphatases. In the present study, we show that PRL-3 may indeed be induced in OC cells by laminin, and that this induction is mediated, at least partially, via activation of PLD.

## 5. Conclusion

In contrast to previous studies indicating that PRL-3 is a marker of more aggressive clinical course, our data show that the clinically relevant PRL members in metastatic OC are PRL-1 and PRL-2, both of which are associated with less aggressive disease. These data suggest that PRL members may have different cellular functions in OC compared to other cancers, which are yet to be characterized. The upregulation of PRL-1 in effusions compared to solid lesions provides further evidence of the unique biology of OC cells at this anatomic site.

## Figures and Tables

**Table 1.** Clinicopathologic data of the study cohort.

**Parameter**		**Material (Number of Patients)**		
		**Effusions (90)**	**Primary (57)**	**Metastasis (27)**
***Age***	Mean (range)	61 (34–79)	60 (31–84)	63 (47–80)
**FIGO Stage**	I	0	4	2
	II	1	5	0
	III	46	27	16
	IV	43	13	9
	NA a	0	8	0
**Grade**	I	9	9	1
	II	21	15	5
	III b	51	26	20
	NA	9	7	1
**Residual Disease**	≤1 cm	32	34	14
	>1 cm	44	14	13
	NA	14	9	0
**Histology**	Serous	75	40	19
	Mucinous	1	0	0
	Clear cell	4	3	2
	Endometrioid	1	7	3
	Mixed epithelial	4	3	0
	Undifferentiated	2	0	3
	NA	3	4	0

a Not available;

b including 9 clear cell carcinomas.

**Table 2.** Primer sequences.

**mRNA**	**Primer Pairs**	**Product Size (bp)**	**Reference**
*PRL-1*	sense: 5′-GACCTGGATGGGGTAAACCT-3′	283	[23]
	Antisense: 5′-TGTGACTTCCACAGGAGCTG-3′		
*PRL-2*	Sense: 5′-TTTCCCATCACACTCACACG-3′	352	[23]
	Antisense:AACACAAGGCACTGCAACAC-3′		
*PRL-3*	Sense: 5′-AGCCCCGTACTTCTTCAGGT-3′	198	[31]
	Antisense: 5′-GGGACTTCTCAGGTCGTGTC-3′		
*28s*	Sense: 5′-GTTCACCCACTAATAGGGAACGTGA-3′	200	[24]
	Antisense: 5′-GGATTCTGACTTAGAGGCGTTCAGT-3′		

**Table 3.** Cycle parameters.

**Gene**	**Heating**	**Denaturation**	**Annealing**	**Extension**	**No. of Cycles**
*PRL-1*	94 °C, 5 min	94 °C, 30 s	60 °C, 30 s	60 °C, 30 s	32
*PRL-2*	94 °C, 5 min	94 °C, 30 s	60 °C, 30 s	72 °C, 30 s	32
*PRL-3*	94 °C, 5 min	94 °C, 30 s	58 °C, 1 min	72 °C, 90 s	32
*28s*	94 °C, 5 min	94 °C, 15 s	63 °C, 20 s	72 °C, 10 s	31
